# Sweetening K-channels: what sugar taught us about permeation and gating

**DOI:** 10.3389/fmolb.2023.1063796

**Published:** 2023-04-14

**Authors:** David Naranjo, Ignacio Diaz-Franulic

**Affiliations:** ^1^ Instituto de Neurociencia, Facultad de Ciencias, Universidad de Valparaíso, Valparaíso, Chile; ^2^ Center for Bioinformatics and Integrative Biology, Facultad de Ciencias de la Vida, Universidad Andrés Bello, Santiago, Chile; ^3^ Centro Interdisciplinario de Neurociencia de Valparaíso, Facultad de Ciencias, Universidad de Valparaíso, Valparaíso, Chile

**Keywords:** Kv-channels, osmotic agent, volumetric work, diffusion limited current, single channel conductance, voltage gated transition, streaming potential

## Abstract

Because they enable for the modification of both viscosity and osmolarity, sugars have been used as a biophysical probe of voltage-gated K-channels for a while. Viscosity variations made it possible to measure the pore sizes in large and small conductance K-channels using techniques similar to those used in the 1980s to study the gramicidin A channel. These analyses led to the finding that the size of the internal mouth appears to be the primary cause of the conductance differences between Shaker-like channels and large conductance BK-channels. As an osmotic agent, adding sugar unilaterally causes streaming potentials that indicate H_2_O/K^+^ cotransport across the BK-channel pore. Osmotic experiments on Shaker K-channels suggest that the pore gate operation and the slow inactivation displace comparable amounts of water. Functionally isolated voltage sensors allow estimation of individual osmotic work for each voltage sensing charge during voltage-activation, reporting dramatic internal and external remodeling of the Voltage Sensing Domain´s solvent exposed surfaces. Remarkably, each charge of the VSD appears to take a unique trajectory. Thus, manipulation of viscosity and osmolarity, together with 3D structures, brings in solid grounds to harmonize function and structure in membrane proteins such as K-channels and, in a wider scope, other structurally dynamic proteins.

## 1 Introduction

The advent of membrane protein crystallography first, and the shattering irruption of single particle cryogenic electron microscopy later, has allowed us to understand with an unprecedented level of detail the structural determinants of gating, permeation, and inactivation of K-channels, subjects that are covered in other reviews of this series. In this section, we briefly discuss the use of sucrose as an osmotic and viscosity agent in order to explore the functional architecture of voltage-gated K-channels during gating and K-permeation. We also discuss how these functional data complement the structural ones.

### 1.1 Sugar, sugar: sucrose is a hygroscopic disaccharide

The most popular home sugar is sucrose. We learned from our household experience that sucrose’s solubility in water could be quite high, making it suitable for use as a food preservative. Glucose and fructose molecules are joined by an ether bond between C1 and C2 on the glucosyl and fructosyl units, respectively, to form this disaccharide, which is a naturally occurring compound in plants. Its chemical structure is C_12_H_22_O_11_. It can make hydrogen bonds with water thanks to its eight hydroxyl groups, which could be the cause of its high solubility (Imberti et al., 2019). Sucrose in aqueous solution integrates into the water hydrogen bond network, bonding with water and other sucrose molecules, resulting in a more compacted solution (Starzak et al., 2000). Anyone who has performed a sucrose gradient centrifugation has probably noticed that the solution densities are greater and significantly more viscous (a ∼ 1M sucrose aqueous solution is ρ ∼1.15 gr/ml with a water-relative viscosity η/η_w_ ∼ 4.3) (Hofmann, 1977). A molecular explanation for macroscopic viscosity is that the hydrogen bond network couples molecules to each other, so if two neighboring fluid layers move at different velocities, each tends to drag the other. The dragging forces will tend to dissipate the velocity gradient within the liquid (Rha, 1975). Thus, a diffusing ion, moving under an electric force, will push a wider layer of solvent as if its hydrodynamic dimension were larger. By adding sucrose to the solution to reduce ion mobility, it is possible to find the experimental conditions needed to measure diffusion-limited ion transport across K^+^ channels (Section 2). A unilateral addition of sucrose at one side of an ion channel may drag water and ions from the hypoosmotic side across the channel´s pore. This stream creates a charge separation known as streaming potential that reveals the number of water molecules going across the channel with each permeant ion (Section 3). Also, when sucrose is at a high concentration, any protein conformational change that captures or surrenders water molecules from the solution must negotiate with this hygroscopic solute. Thus, the osmotic pressure has been a tool to assess the physical extension of K-channel gating-related conformational changes (see Section 4).

## 2 Sugar in the opening: the size of the internal entrance determines single channel conductance

The exquisite selectivity of the K-channels for K^+^ over Na^+^ attracted the attention of membrane biophysicists from the start. Considering a difference of ∼0.4Å in their Pauling radius, they exhibited a remarkable permeability ratio of P_Na+/_P_K+_<0.01. The permeability ratios for alkali cation values obtained in the K-conductance of the myelinated nerve from frog (P_Tl+_>P_K+_>P_Rb+_>P_NH+_>>P_Li+_∼P_Na+_∼P_Cs+_) were then explained in terms of the binding equilibrium contributed by steric, solvation, and electrostatic influences (Eisenman, 1962; Hille, 1973). Later on, in the giant squid axon, the high selectivity was explained in terms of size selection by a rigid structure, possibly lined by carbonyl groups, that could precisely accommodate dehydrated or partially dehydrated K^+^ ions but not Na^+^ ions (Bezanilla and Armstrong, 1972). Blockade experiments, best known for the *foot in the door effect*, of intracellular organic cations, crafted an overall shape where the permeation pathway begins in a wide intracellular entrance, followed by the rigid selectivity filter (Bezanilla and Armstrong, 1972; Coronado and Miller, 1982; Latorre and Miller, 1983). Those architectural anticipations of the permeation system turned out to be accurate when the Mackinnon group served at the table the atomic coordinates of KcsA, a prokaryotic potassium channel from the soil bacterium *Streptomyces lividans* at 3.2 Å (Doyle et al., 1998). The channel structure, in a closed or near-closed state, showed a ∼18 Å-long pore with an intracellular bundle crossing and locking a presumably aqueous wider inner cavity. Such cavity possibly decreases the energetic penalties of placing a charged particle at the membrane center (Parsegian, 1975; Roux and MacKinnon, 1999). Although the resolution of that structure prevented direct assignment of the carbonyl’s oxygen atoms, the authors placed them facing the passing ions. (Heginbotham et al., 1994). Shortly after, the same group showed the carbonyl atoms acting as water surrogates, allowing them to strip away the K^+^ from their hydration cage with minor energy costs (Morais-Cabral et al., 2001; Zhou et al., 2001). The multi-ion nature of the pore, first anticipated by Hodgkin and Keynes (1955), was also confirmed in these structures. The electron density showed five equally populated binding sites: four in the selectivity filter and one in the inner cavity. The selectivity filter would be occupied in two energetically equivalent single-file configurations with 2W:2K stoichiometry, interchanging in a concerted fashion. In both configurations, two K^+^ ions alternate with two water molecules (see below (Alcayaga et al., 1989; Morais-Cabral et al., 2001; Zhou et al., 2001)).

Despite having identical selectivity filters, unitary conductance in small and large conductance K-channels can differ by 10-100-fold (Eisenman et al., 1986; Heginbotham and MacKinnon, 1993; Naranjo et al., 2016). The above question hovered in the field for a long time. Only after the structure of the Kv1.2, a small conductance channel (10 pS) was determined in 2005 (Long et al., 2005), the bacterial MthK, and the *Aplysia* Slo1.2 large conductance channels (250-300 pS) came to light, was there the possibility to look into the physical support for the conductance differences (Jiang et al., 2002; Tao et al., 2017). Sugars played a significant role in the development of a satisfactory conduction model that accounted for the functional differences. But first, a little bit of history.

**FIGURE 1:**
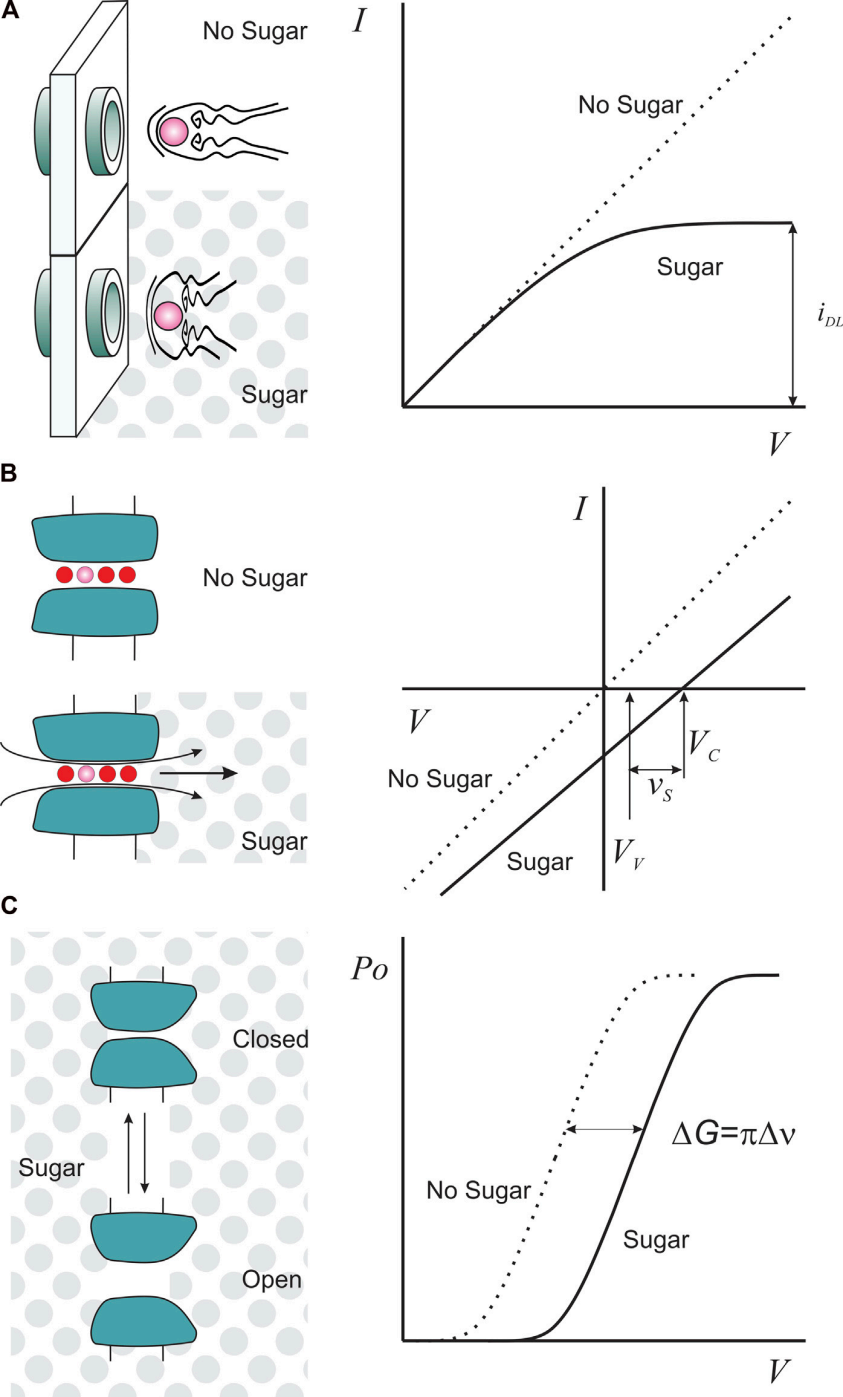
Sugars as functional tools for K-channels. **(A)**. Sugar (gray dots) as a viscosity agent reduces ion diffusional mobility such that it is possible to obtain saturating currents, *i_DL_*, at lower voltages (right). **(B)**. Unilateral sugar application, as an osmotic agent, produces a streaming potential with a magnitude proportional to the number of water molecules (red circles) escorting one permeant ion (pink). *V_C_* is the ion channel reversal potential in the presence of sugar, and *V_V_* is the valinomycin potential, measured to correct for activity changes in sugar solution (see main text). **(C)**. Sugar as an osmotic agent imposes an additional work (ΔG) to the voltage-dependent channel opening (Po) if the conformational change exposes or hides an inaccessible sucrose volume (Δν), equating volumetric and electric works.

The Gramicidin A channel is a linear decapeptide with antibiotic capabilities that, in its dimeric form, induces discrete ion conduction events that saturate at high electrolyte concentrations (Hladky and Haydon, 1970; Veatch and Stryer, 1977). Interestingly, the ionic selectivity of the Gramicidin A channel mirrors the ion mobilities in solution, which, at first glance, suggests a water-filled hole (Myers and Haydon, 1972). But both the small diameter of the conduction pathway (4 Å) and streaming potential measurements are incompatible with that model, suggesting instead a single-file conduction mechanism (Rosenberg and Finkelstein, 1978b; Levitt et al., 1978). In this scenario, diffusionally limited access to the channel pore would strongly influence the association rate of the permeant ions. How to estimate the size of the pore entrance? Imagine a pore embedded in the plasma membrane bathed in a saline solution, where the ion flux through it is driven by the transmembrane potential (Figure 1A). If we can increase the transmembrane potential in an unlimited way, inevitably, at some point the ion flux will be limited by the arrival of the ions at the channel entrance, becoming voltage independent (Lauger, 1976; Figure 1A). Eq. 1 describes this diffusion-limited current, *i*_*DL*_, as:

$$i_{DL}=2\pi ze_{o}r_{C}Dc$$ (1)

where *z* is the valence of the permeant ion, *e*_*o*_ is the value of an elementary charge, *r*_*C*_ is the radius of capture, *D* is the diffusion coefficient, and *c* is the bulk concentration of the permeant ion expressed as ions/cm^3^. Assuming that the channel pore is a cylinder with neutral walls and the approaching ions are point charges, *r*_*C*_ is the pore size required to capture enough ions to account for *i*_*DL*_. With known *z*, *e*_*o*_, *D,* and c, *i*_*DL*_ provides a unique estimation of the pore-opening dimensions. To be sure that the currents saturation is of diffusional origin, its amplitude should be proportional to the concentration and diffusion coefficient of the permeant ion. Andersen experimentally decreased *D* in four different ways: by substitution of H_2_O for D_2_O, with temperature changes, and with the addition of sucrose or glycerol to increase the viscosity of the aqueous solution (because *D* is inversely proportional to η). However, *r*_*C*_ is an operational parameter because ions are not point charges and are not alone. Ions have a finite radius and usually travel with a gang of several hydration water molecules attached with different degrees of adherence. Then, the radius of capture does not, and should not, coincide with the physical dimensions of the pore. A better description of *r*_*C*_ is the difference between the physical pore radius and the hydrodynamic ion radius (Ferry, 1936; Andersen, 1983; Brelidze and Magleby, 2005). Then:

$$r_{C}=r_{p}-r_{ion}$$ (2)

where *r*_*P*_ is the pore entrance radius and *r*_*ion*_ is the hydrodynamic radius of the ion in an aqueous solution. This equation states that the total number of effective collisions for a point, or spherical, charge is identical for equal radial deviations from the central trajectory.

The results obtained in 0.1M salt solution for Gramicidin A converged on capture radii of ∼0.2 Å for K^+^, Rb^+^ and Cs^+^, that having in mind structural estimations of *r*_*P*_ ∼2 Å (Urry et al., 1971; Koeppe et al., 1979), would make a reasonable use of Eq. 2 only if we consider the naked ionic radii for these ions (1.4-1.7 Å). But we all know that metal cations do not go around naked. With a hydrodynamic radius contributed by ∼6-8 water hydration layer, *r*_*ion*_ = 3.6-4.0 Å, the pore radius *r*_*p*_ would be ∼4 Å, doubling the structural estimation. The causes of such discord may be several, such as a lower effective sucrose concentration in the vicinity of the membrane or a binding reaction that departs from models that envision ion access to the pore as a simple collision with a spherical surface (Andersen, 1984). However, the important point is that the capture radius is perhaps the most realistic functional estimate of the behavior of an ion in the vicinity of the channel, and as we will see later, under certain conditions, it is also able to provide unique information about the pore architecture.

### 2.1 Functional estimations agree with the structure of the K-channel inner pore dimension

The advent of X-ray crystallography shed light on the engine room of K^+^ channel conduction, explaining both ion selectivity and high permeation rates. However, despite the high conservation of their signature sequence, the 10-to 100-fold difference in their single channel conductance leaves open the question about the structural diversity and the conductance determinants of K-channels.

The faster blocking/unblocking rates of the BK-channel (the calcium and voltage-sensitive large conductance channel) compared with previously characterized small conductance K-channels pointed to different sizes in the inner vestibules as one possible feature accounting for different ion transport rates (French and Shoukimas, 1981; Taglialatela et al., 1991; Choi et al., 1993; Li and Aldrich, 2004). In addition, trapping Shaker K-channels in the open conformation by metal bridge formation at the permeation pathway suggested the existence of a ∼8-9Å wide inner entrance (Webster et al., 2004). The lack, by then, of a structure for BK channels led Magleby´s group to use viscosity agents to probe the pore cytosolic dimensions. To provide the electroneutral surface that theory requires, the limiting currents had to be measured in a BK channel variant lacking a ring of eight negatively charged residues at the cytosolic side. These charges could raise the local [K^+^], distorting the measurement (Brelidze et al., 2003). The diffusion-limited current of the neutral BK variant was ∼5 pA in 150 mM [K^+^] +2M sucrose, which considering a diffusion coefficient for K^+^ 0.25 × 10^−5^ cm^2^/s renders an effective capture radius of 2.2Å. Interestingly, the dimensions for the BK channel inner vestibule made sense with the 18-20Å-wide pore revealed by the structure of the Archaean Calcium-gated K-channel MthK, obtained from *Methanothermobacter thermautotrophicus* and crystallized by MacKinnon’s group in 2002 (Jiang et al., 2002). Thus, despite being distantly related to BK in evolutionary terms, the Ca^2+^-dependence, the presence of the inner negatively charged ring, and the BK-channel radius of capture were also congruent with the pore dimensions of MthK (Jiang et al., 2002; Brelidze and Magleby, 2005; Shi et al., 2011). The BK channel structure from *Aplysia californica* was resolved 15 years later, showing a 15Å-wide pore, in general agreement with estimations based on the radius of capture (Tao et al., 2017).

In our lab, using sucrose to increase the solution viscosity, we determined the diffusion-limited currents in the small conductance Shaker WT, together with the large and intermediate conductance variants P475D/Q. All variants rendered a *r*_*C*_∼0.8Å, which is possible to harmonize with the structure of the, presumably open, Shaker mammalian homologue Kv1.2 if the hydrated potassium ion ranges between 3.8 and 4.2Å (Diaz-Franulic et al., 2015; Moldenhauer et al., 2016). Considering that *r*_*C*_ estimates were from experiments in the liquid phase, at room temperature, away from equilibrium, with fully operative friction forces, assuming spheres moving in a continuum, and, on the other hand, K^+^-channel structures were obtained at low temperature, in the solid phase, and in equilibrium, the agreements between functional and structural data are very satisfying.

### 2.2 Inner pore dimensions may determine single channel conductance

Despite the fact that pore dimensions are evidently different between small and large conductance K-channels, their relative contribution to the ion transport rate across the pore remained to be elucidated. A single-point mutation (Pro 475→Asp) at the internal entrance of the low-conductance Shaker K-channel increases unitary currents by 6–8-fold (Sukhareva et al., 2003), which we proposed arose from increased pore occupancy due to the electronegative surface that mutation creates (Moscoso et al., 2012). Such a dramatic increase on the otherwise modest unitary conductance of Shaker led us to wonder what structural features are limiting the ion permeation rate through a small-conductance K-channel.

Using the radius of capture measurements of Shaker and the increased conductance variants P475D/Q (Diaz-Franulic et al., 2015) and considering a resistance of the selectivity filter of 0.9 GΩ we devised a 5-in-series resistance model to frame both internal and external sucrose exposure. Keeping in mind that a 7-fold decrease in solution conductivity in 2M sucrose we proposed that the inner pore, not the selectivity filter, is the largest resistance along the permeation pathway (30 GΩ), followed by the internal access resistance (8 GΩ) and the external access resistance (2GΩ) (Yellen, 1984; Diaz-Franulic et al., 2015). This inner pore resistance was decreased by polar mutations P475D/Q, leading to maximal unitary conductances of 250 pS and 150 pS, respectively. However, even after such a large decrease in the main resistive element of the channel pore, its maximal ion transport rate is still only 1/3 of that of BK channels obtained at saturating K^+^ concentrations (600 pS). The conductance gap between small and large conductance K-channels is then filled by a larger internal access resistance that arises from a smaller pore size (Diaz-Franulic et al., 2015).

Analysis of the MthK and Kv1.2-2.1 structures in light of the aforementioned *r*_*C*_ rendered hydrated K^+^ radii of 3.1–3.7 Å and 3.6–4.4 Å, respectively, is in nice agreement with previous determinations (Enderby, 1995; Glezakou et al., 2006; Mancinelli et al., 2007), validating the use of the radius of capture as an accurate and effective method to scrutinize the structural determinants of the conduction process across biological membranes.

## 3 Sugar on the side: electroosmosis reveals the water gang traveling with K^+^ across the pore

As mentioned above, the pore in the KscA structure crystallized in KCl clearly showed ∼5 fully occupied electronic densities in single file formation, four in the selectivity filter and one in the cavity (Doyle et al., 1998; Zhou et al., 2001). Because the densities located in the selectivity filter could represent occupation by either K^+^ or H_2_O, based on, let´s say, common sense, K^+^ ions in the selectivity filter were assigned to be, ∼7Å apart, separated by one water molecule ∼3.5Å away. Closer proximity of two naked K^+^ would be energetically forbidden. Considering streaming-potentials measurements on BK-channels and energetic calculations on KcsA, a 1:1 K^+^/H_2_O (K/W) transport stoichiometry was suggested (Alcayaga et al., 1989; Morais-Cabral et al., 2001). Thus, the four binding sites occupancy should alternate between two energetically equivalent, and concertedly inter-exchanging, sequence configurations: WKWK or KWKW (Morais-Cabral et al., 2001). Such a model predicted the basic aspects of ion conduction, such as high K^+^ transport rates, K^+^ dependency and selectivity (Morais-Cabral et al., 2001). This permeation mechanism reigned for about a decade, until Molecular Dynamic simulations with applied transmembrane potential to produce permeation events challenged this vision, suggesting that such a permeation mechanism is not unique in KcsA and nor generalized to the different K-channels. Defying common sense, permeation events could be induced by direct K^+^-K^+^ interaction without intervening water; worse, three or four K^+^ could normally occupy the selectivity filter sites, totally excluding water, and with not equally distributed binding probabilities (Furini and Domene, 2009; Jensen et al., 2010; Jensen et al., 2013; Kopec et al., 2018; Domene et al., 2021). Thus, conduction in K-channels seemed to be fairly anarchic and distinct across K-channels, where the transport K:W stoichiometry is larger than 1:1 and often with no water being co-transported. In addition, analysis of experimental 2D IR spectra of synthetic KcsA supports mechanisms of ion permeation with and without co-permeating water (Kopec et al., 2018).

Clearly, new experimental data are necessary to assess the K:W transport stoichiometry on different K-channels. Beside Alcayaga et al. (1989), to our knowledge, no other electrophysiological attempt has been made to determine the number of water molecules escorting K^+^ ions across the K-channels pore. Here we superficially review how to calculate the K:W transport stoichiometry from streaming potential measurements. For more detail, see Rosenberg and Finkelstein (1978a).

Consider a membrane with cation-selective channels that separates two compartments containing a diluted monovalent salt (let´s say 50 mM, much less than the sugar concentration). The ion channel pore is long enough to host a number of water molecules and one ion in single file (Figure 1B). When challenged with a unilateral addition of 1 M sugar to impose an osmotic gradient, water moves from the hypotonic side, dragging the ions dwelling in the pore. This extra charge-flow creates an additional electrical transmembrane potential. Equilibrium is reached when the new transmembrane potential (the streaming potential, *v*_*s*_) completely counterbalances the water differential potential energy (Rosenberg and Finkelstein, 1978a). A pore lacking ions would only move water, and one lacking water would be undisturbed by the osmotic gradient. In these two cases, no streaming potential would result. As a result, a non-zero *v*_*s*_ necessitates the coexistence of water and ions in a single file within the pore. The streaming potential at low ionic concentration for a channel hosting one ion would be:

$$v_{s}\approx N\frac{RT}{F}\frac{{\varphi_{s}n}_{s}}{n_{w}}$$ (3)

where *N* is the number of H_2_O per ion, *R,T* and *F* have their usual meanings, *n*_*s*_ and *n*_*w*_ are the molar concentration of sucrose (∼1M) and water, respectively. *φ*_*s*_ is the molal osmotic coefficient. In a voltage clamp experiment, *v*_*s*_ could be, in principle, measured directly as the K-channel reversal potential (*V*_*C*_) Figure 1B). However, it is necessary to introduce a correction for the effect of sugar on the activity of the ionic species. This correction is usually carried out by adding to both sides of the membrane an excess of valinomycin, a dehydrated-K^+^ selective ionophore, and measuring the ionophore-induced new reversal potential (*V*_*V*_) (Rosenberg and Finkelstein, 1978a; Alcayaga et al., 1989). Thus, as Figure 1B shows:

$$v_{s}=V_{C}-V_{V}$$ (4)

When, *φ*_*s*_ *n*_*s*_ *≈* 1 (sucrose around 1M) and room T^o^, Eq. 3 reduces to *v*_*s*_ *≈ N*x0.46 mV. For gramicidin A channel in 10 and 100 mM NaCl, KCl or CsCl solutions, *v*_*s*_ *≈* 3 mV/Osm per kg, pointing to a W:ion stoichiometry of 6-7:1, value in agreement with recent MD simulations (Rosenberg and Finkelstein, 1978a; Paulino et al., 2020). The hemocyanin and the sarcoplasmic reticulum K^+^ channels in sugar or urea gradient showed *v*_*s*_ *≈* 1.2 and 1.1 mV/Osm per kg, respectively, pointing to two to three coupled water molecules (Cecchi et al., 1982; Miller, 1982). In the probably most relevant finding for the discussion here, the skeletal muscle BK-single channel recordings in artificial lipid bilayer exposed to 2M unilateral sucrose exposure gave *v*_*s*_ *≈* 0.85 mV/Osm per kg, pointing to a 1-2:1 W:K stoichiometry in physiological solutions (Alcayaga et al., 1989). Considering that BK-channels are multi-ion channels, these results are in good agreement with, and possibly inspired, the classic 2W:2K permeation mechanism (Morais Cabral et al., 1998; Morais-Cabral et al., 2001; Zhou et al., 2001).

It is worth noting that with a gradient of 2 Osm/kg, Alcayaga et al. (1989) obtained *v*_*s*_ *=*1.86 ± 0.3 mV. This small number is reliable thanks to the exceptional signal-to-noise ratio offered by the BK-channel. Two additional criteria strengthen this type of determination: First, the streaming potential should be proportional to the osmotic gradients (Cecchi et al., 1982; Miller, 1982). Second, in the Alcayaga et al. (1989) measurements, *v*_*s*_ decreased significantly as the K^+^ concentration was symmetrically raised from 20 to 500 mM, a concentration range in which BK single channel conductance grows ∼4 fold (Eisenman et al., 1986). These two determinations reported strong functionally congruent changes in the K^+^ occupancy in the BK-channel, providing a critical support for water being co-transported in the BK channel. To replicate these experiments in small/intermediate conductance Kv-channels, where, according to molecular simulations, water is severely excluded, would be exceedingly difficult to perform. Increasing the osmolyte by two or fourfold to circumvent this difficulty, could enable the identification of conduction modes that exclude water or have a low W:K stoichiometry. Because of their high conductance, the interesting cases of KcsA and MthK channels, among others, could still offer a manageable signal-to-noise ratio to perform osmotic measurements that could contribute to clarifying the W:K stoichiometric discord in K-channel permeation (Naranjo et al., 2016).

## 4 Sugar in the gating: VSD activation does a large osmotic work

Voltage-gated activation is the most distinctive conformational change in voltage-gated K-channels (Kv). This structural change allows the channel to open, conducting K^+^-ions. Kv channels have a dedicated Voltage-Sensor-Domain, VSD, which undergoes a large conformational change in response to changes in the transmembrane electric field. Over the past 25 years, the nature and extent of these conformational changes have been intensively debated. This section will exclusively discuss the structure-function lessons we learned using sucrose as an osmotic agent.

Consider a protein having two conformations. The addition of an osmotic agent to the protein surroundings will shift the equilibrium in favor of the conformation that exposes less hydrated surfaces that are not in contact with the osmolyte (Figure 1C). Thus, the impact of the osmolyte on the free energy of the conformation change makes it possible to estimate its extension if it involves fluctuations in the protein’s level of hydration. This estimation is reported as a change in volume exposed to the solvent but buried from the osmolyte (Figure 1C). This technique was initially used to infer the pore aqueous volume variations on the opening of the reconstituted mitochondrial voltage-gated porin (VDAC) during activation (Zimmerberg and Parsegian, 1986).

Examining activation-induced conformational changes in TTX-poisoned squid giant axons was the first application of this technique to voltage-gated potassium channels. Sucrose osmotic effects revealed a voltage independent increment of sucrose inaccessible aqueous volume of 1500Å^3^ (Zimmerberg et al., 1990). With the development of molecular cloning and heterologous expression of channels, experiments could be carried out under circumstances where the targeted protein is responsible for most of the signal. As a result, Shaker K-channels subjected to a moderate unilateral increase in external osmotic pressure showed only a slight effect on their ability to activate, indicating, either, little external physical changes to the water-filled volume or that modifications with similar glucose accessibility occurred. Therefore, rather than the voltage-sensing conformational change, the pore opening may have been primarily responsible for the volume variation. Similar outcomes were obtained from tests employing Kv1.4 expressed in *Xenopus* oocytes, but with an unusual twist. The estimated change in cytosolic volume during activation was around three times bigger than the estimate for giant squid axon K-channels and comparable to that of slow-type inactivation (Jiang et al., 2003). These findings showed that, similar to Shaker and Kv1.2, the activation gate and, at least, one of the slow inactivation gates had a large structural overlap, as indicated by the internal TEA interference with both gates (Gonzalez-Perez et al., 2008; Suarez-Delgado et al., 2020). Other slow inactivation gate (C-type) that appears to be toward the external pore opening (Choi et al., 1991; Oliva et al., 2005), seems to be sensitive to the external osmolarity. Ostemeyer et al. (2013) conducted extensive molecular simulations of KcsA channels, finding that, in the slow inactivated state, the selectivity filter is stabilized by three water molecules situated behind the pore-lining residues. Removal of these waters helps the filter to assume a conductive state. Accordingly, electrophysiological experiments revealed an accelerated recovery from inactivation in 2M external sucrose, suggesting that the conductive conformation of the selectivity filter is favored when sucrose grabs nasty inactivation waters from the channel (Ostmeyer et al., 2013). Thus, water may play a critical role in the channel’s conformational equilibrium.

Conceptually speaking, the isolation of the VSD from the rest of the protein yields a simpler system and more straightforward data interpretation of volumetric changes. However, only when it became evident that the VSD activation implied an 8–15 Å displacement of the voltage sensing charges did it become necessary to explore the volumetric extent of the VSD conformational change (Vargas et al., 2012). But, as mentioned above, such conformational change was, if not undetectable, at least obscured by the one occurring at the pore gate (Zimmerberg et al., 1990; Starkus et al., 1995; Jiang et al., 2003).

### 4.1 The VSD osmotic work is measured by an osmotic induced shift in the Q-V relation

Let’s assume that the VSD transits between a few conformational states, but dwells mostly in two: the resting (R) and active (A) conformations. If that transition involves a change in the sucrose-inaccessible aqueous volume in hyper- or hypotonic conditions, additional osmotic work must be performed. The total free energy (*ΔG*) of the transition is the sum of the conformational change free energy (*ΔG*_*o*_) plus the electrical and osmotic work.

$$∆G=∆G_{o}+\pi ∆\nu -zFV$$ (5)

*π* is the osmotic pressure, and Δν is the variation of solute-inaccessible aqueous volume, z is the charge equivalent mobilized during activation, F is the Faraday constant, and V is the applied voltage. The negative sign in the electrical term specifies that positive voltages favor the active conformation.

Shaker-V478W is an ideal variant to record isolated VSD activity. The 478-tryptophan side chains form a hydrophobic plug that prevents the inner gate pore opening (and its related osmotic work) (Kitaguchi et al., 2004). In this constitutively closed Shaker variation, each VSD autonomously transits back and forth between R and A since it lacks the concerted transition into the stable open pore conformation (Zagotta et al., 1994). *Gating currents* measurements are the proper way to measure isolated VSD activity, which represents the recording of the movement of the voltage sensing charges across the transmembrane electric field (Armstrong and Bezanilla, 1973; Keynes and Rojas, 1974; Diaz-Franulic et al., 2018). Then, the osmotically induced shift in the Q-V relation reports the volumetric changes in the voltage sensor. Thus:

$$\Delta \nu =\frac{zF\Delta V_{M}}{\Delta \pi }$$ (6)

In which *Δν* is the volumetric change per mol of VSD, Δπ the change in osmotic pressure, *V*_*M*_ is the median voltage at which both states R and A are equally populated (*ΔG* = 0), and *ΔV*_*M*_ is its osmotic induced shift (Chowdhury and Chanda, 2012). The osmotic pressure, defined as Δπ = Δc*RT,* with Δc being the sucrose increase over standard recording conditions and R and T are the gas constant and temperature, respectively. A symmetrical sucrose concentration *Δ*c = 2M (the equivalent of *Δπ* ∼50 Atm) produced a *ΔV*_*M*_ = +8 mV, which, with a z∼3, corresponds to ∼900 Å^3^ increase in sucrose-excluded aqueous volume in each VSD (Diaz-Franulic et al., 2018). The unilateral sucrose application showed that most of the volumetric growth is external. Although this volume change is modest in comparison to that of the pore opening found by (Jiang et al., 2003), it would be comparable if all four subunits were included. We may speculate that in the fully functional wt-Shaker these volumetric changes were masked by other vectorially opposed water displacements, in other channel domains. Considering that flexibility of the S4-S5 linker significantly affects channel gating (Sands et al., 2006; Balleza et al., 2015; Liin et al., 2015) and that the presence of internal sucrose slows down channel opening and closing (Zimmerberg et al., 1990) it cannot be excluded that some osmotic work needs to be performed at the pore opening electromechanical coupling interface. Additionally, the VSD volumetric change could result from even larger separate, and mutually canceling internal and external volumetric displacements.

Assuming that the net volumetric work of the voltage sensor should be the aggregated work of the individual voltage sensing arginine residues in S4, they were individually neutralized. Then, the osmotic work brought about by a given arginine should be the osmotic work of the native Shaker-V478W minus that of the given arginine-missing Shaker-variant. Additionally, the individual contribution of each S4 arginine can be estimated from the linear combination of all 4 single arginine missing Shaker-variants. Remarkably, both methods gave similar results with either bilateral or unilateral sucrose exposure.

R1 and R4 were exclusively osmotically sensitive on the external and internal faces, respectively. Meanwhile, R2 and R3 were sensitive to osmotic stress in both faces but with opposite sensitivities. Thus, R2 and R3 possibly perform vectorially opposed volumetric works, equivalent to the disappearance of a ∼1,200 Å^3^ internal puddle and the formation of a ∼1900 Å^3^ external one (Diaz-Franulic et al., 2018). The volumetric work of R1+R2+R3+R4 is ∼1000 Å^3^ which compares well with the ∼900 Å^3^ per VSD of the native Shaker-V478W. The VSD net volumetric work could result from an inward displacement of the septum or the charge transfer center or a transporter-like conformational change (Ahern and Horn, 2004; Chanda et al., 2005; Li et al., 2014).

The linear additivity of the individual voltage-sensing arginines on the total VSD volumetric work leads to three main conclusions that can be drawn mostly from the osmotic work measurements: 1) The entire structural volumetric work of the VSD can be accounted for solely by the volumetric work caused by R1-R4 rearrangements; 2) The hydration landscape for each voltage-sensing residue is completely different, implying separate activation trajectories for the voltage sensing arginines or the absence of a unique charge transfer pathway; 3) The VSD is a structurally robust protein domain surrounded by a very malleable lipid and electrostatic environment that retains its structure and function despite mutations in R1-R4, a surprising result given how well conserved R1-R4 are (Gonzalez-Perez et al., 2010; Diaz-Franulic et al., 2018; Naranjo, 2022).

## 5 Concluding remarks

The use of sucrose is far from being limited to the study of ion conduction and the gating of Kv channels. It has been used in the study of hemoglobin, allowing us to observe that their tense and relaxed conformations in their deoxy and oxygenated states, respectively, have significant changes on their solvent exposed surfaces, suggesting a role for hydration in the protein conformational dynamics (Shaanan, 1983). After assessing the effect of several organic osmolytes on the O_2_ binding curves, Colombo et al. concluded 50 to 70 water molecules are gained during the deoxy to oxy transition of the Hb tetramer (Colombo et al., 1992) in agreement with previous determinations obtained with the method of modulation of entropy by alcohols (Bulone et al., 1991). De Cristofaro et al. performed osmotic stress experiments using sucrose to address the role of water in the thrombin-thrombomodulin complex formation, concluding that ∼35 water molecules are released into the bulk solution during the process (De Cristofaro et al., 1995). Sucrose has also contributed to understanding the underlying mechanism of the unparalleled temperature dependence of thermoTRP channels. Diaz-Franulic el at. (2020), showed that the carboxy terminal coiled coil of the TRPM8 channel was not only required for making the channel cold sensitive but also that it experienced a significant increase in its solvent-inaccessible aqueous volume of ∼1500Å^3^ upon cold exposure (Diaz-Franulic et al., 2020), which was compatible with the channel structure determined by Cryo-EM (Yin et al., 2018). Considering these findings, it seems plausible that the steep temperature dependence of channel gating arises from a folding/unfolding reaction at the carboxy terminus domain, which increases its molar heat capacity in the unfolded state (Clapham and Miller, 2011; Diaz-Franulic et al., 2020; Diaz-Franulic et al., 2021). Overall, the above studies highlight the importance of considering hydration not only as a major player in protein folding but also as an agent capable of shaping the conformational landscape governing their biological activity.
